# Primary Sjögren’s syndrome presenting with non-diffuse membranoproliferative glomerulonephritis-like lesions in cryoglobulinemic glomerulonephritis: a case report

**DOI:** 10.3389/fimmu.2025.1610017

**Published:** 2025-05-26

**Authors:** Zhi-Yu Duan, Guang-Yan Cai, FengKun Chen, XuanLi Tang, Xu Zhang, YaLi Ren, Yan Song

**Affiliations:** ^1^ Department of Nephrology, First Medical Center of Chinese People's Liberation Army (PLA) General Hospital, National Key Laboratory of Kidney Diseases, National Clinical Research Center for Kidney Diseases, Beijing, China; ^2^ Beijing Key Laboratory of Kidney Diseases Research, Department of Nephrology, First Medical Center of Chinese PLA General Hospital, National Clinical Research Center for Kidney Diseases, Beijing, China; ^3^ Department of Nephrology (Key Laboratory of Kidney Disease Prevention and Control Technology), Hangzhou Traditional Chinese Medicine (TCM) Hospital Affiliated to Zhejiang Chinese Medical University, Hangzhou, China; ^4^ Renal Division, Department of Medicine, Institute of Nephrology, Peking University First Hospital, Peking University, Beijing, China

**Keywords:** primary Sjögren’s syndrome, cryoglobulinemia, cryoglobulinemic glomerulonephritis, membranoproliferative glomerulonephritis, case report

## Abstract

**Background:**

In primary membranoproliferative glomerulonephritis (MPGN), mesangial and capillary proliferation manifests as a diffuse, spherical change; in secondary MPGN, lesions are predominantly focal and segmental.

**Case presentation:**

A 52-year-old woman with a 2-year history of primary Sjögren’s Syndrome (pSS) and no prior renal involvement developed fever, nephrotic syndrome, and acute kidney injury with oliguria after a pulmonary infection. Her highest recorded serum creatinine level was 327.3 µmol/L, and the lowest serum albumin level was 22.1 g/L. Laboratory findings included an antinuclear antibody titer of 1:320, anti-SSA/52KD antibody positivity, complement C3 of 0.468 g/L, complement C4 of 0.0107 g/L, and rheumatoid factor (RF) 678 IU/mL. The highest 24-hour urinary protein quantification reached 9.78 g/24h. After anti-infective treatment, urine output gradually increased, and edema resolved. Cryoglobulin testing showed type II cryoglobulin positivity. Light microscopy revealed MPGN-like lesions in 66.7% of glomeruli and mesangial proliferative glomerulonephritis-like lesions in 25%. Final diagnosis was MPGN. Cyclophosphamide and methylprednisolone were administered. After 30 months follow-up, the patient’s serum creatinine level was 82.1 µmol/L; proteinuria was negative.

**Conclusion:**

This case represents the first reported instance of non-diffuse MPGN with cryoglobulinemic GN secondary to pSS. Infection may serve as a key factor in exacerbating cryoglobulinemia and triggering cryoglobulinemic GN onset.

## Background

The incidence of kidney injury in primary Sjögren’s Syndrome (pSS) ranges from 0.8% to 12.3%; glomerular involvement occurs in 19.1% to 48.5% of cases (1–4). The most common glomerular lesions associated with pSS are membranoproliferative glomerulonephritis (MPGN) and membranous nephropathy (5, 6). In pSS, MPGN is mainly secondary to cryoglobulinemia (6) and classified as immune complex-mediated MPGN (7). In primary MPGN, mesangial and capillary proliferation constitutes a diffuse, spherical change; in secondary MPGN, lesions are predominantly focal and segmental (8). Secondary MPGN, such as type IV lupus nephritis (LN) may present with non-diffuse MPGN-like lesions in class IV segmental and class IV global (9). No reports have documented focal (non-diffuse) MPGN in cryoglobulinemic glomerulonephritis (GN) secondary to pSS. This report describes a case of pSS with cryoglobulinemia leading to cryoglobulinemic GN.

Light microscopy revealed MPGN-like lesions in 66.7% of the patient’s glomeruli; mesangial proliferative glomerulonephritis (MsPGN)-like lesions were present in 25% of glomeruli. Electron microscopy confirmed MsPGN, resulting in a final diagnosis of MPGN. This case provides a pathological diagnosis and management approach for atypical MPGN-like lesions similar to those caused by cryoglobulinemic GN in clinical practice, especially for cases where the diagnosis is inconsistent between light and electron microscopy.

## Case presentation

A 52-year-old woman with a 2-year history of pSS and no prior renal involvement developed cough and sputum in July 2022, with a body temperature of 38.5 °C. Symptoms were accompanied by eyelid edema and hypertension, as well as blood pressure of 182/100mmHg. Chest computed tomography at a local hospital revealed multiple patchy consolidation shadows in both lungs. Laboratory tests showed a serum creatinine level of 94.2 µmol/L and serum albumin level of 34.2 g/L. Additional findings included an antinuclear antibody (ANA) titer of 1:320, complement C3 of 0.468 g/L, complement C4 of 0.0107 g/L, and rheumatoid factor (RF) of 678 IU/mL. Anti-dsDNA and anti-smooth muscle (SM) antibody findings were negative. B-type natriuretic peptide exceeded 35,000 pg/mL. Urinalysis detected proteinuria (2+) and hematuria (2+). The patient received treatment at the local hospital, including anti-infective therapy, anethole trisulfide, and hydroxychloroquine. However, body swelling worsened, urine output decreased, and weight increased by 10 kg within 1 week. Symptoms progressed to nocturnal chest tightness and orthopnea. She was subsequently transferred to our department for hospitalization. On admission, the patient had a body temperature of 38.4 °C and remained in a semi-recumbent position. Auscultation revealed prominent wet rales in both lungs. Laboratory findings included a white blood cell count of 15.15 ×10⁹/L, neutrophil percentage of 89.4%, and hemoglobin level of 98 g/L. Serum albumin was 22.1 g/L, and serum creatinine had increased to 327.3 µmol/L (**Table 1**). C-reactive protein was elevated at 152.11 mg/L, procalcitonin was 0.403 ng/mL, and erythrocyte sedimentation rate was 38mm/h. Further immunological testing showed an ANA titer of 1:80, anti-SSA/52KD positivity (3+), complement C3 of 0.6 g/L, complement C4 of 0.01 g/L, IgG of 3.67 g/L, and RF of 33.8 IU/mL. The Coombs test result was negative. Anti-dsDNA, anti-SM, anti-SSA/60KD, anti-SSB, anti-ribosome, anti-U1RNP, anti-neutrophil cytoplasmic antibodies (ANCA), and anti-glomerular basement membrane (anti-GBM) antibody findings were negative. Blood immunofixation electrophoresis identified monoclonal IgM kappa. Microscopic examination of urinary red blood cells revealed 30–40 per high-power field; 40% exhibited polymorphic morphology, primarily donut-shaped. The 24-hour urinary protein quantification ranged from 7.72 g-9.78 g/24h. Chest computed tomography showed bilateral pneumonia with incomplete airway dilation. Analyses of hepatitis B, hepatitis C, syphilis antibodies, HIV antibodies, severe acute respiratory syndrome coronavirus 2 nucleic acid, influenza A and B virus antibodies, and *Mycoplasma pneumoniae* antibodies all demonstrated negative results. After anti-infective treatment, urine output gradually increased, edema subsided, and body temperature normalized within 3 days.

**Table 1:** Laboratory data.

Variable	Onset	Renal biopsy	Follow-up
Months	0	24	54
Albumin (g/L)	45.2	22.1	43.4
Serum creatinine (μmol/L)	71	327.3	82.1
Serum C3 (g/L)	0.634	0.60	1.04
Serum C4 (g/L)	0.0002	0.01	0.107
ANA (titer)	1:320	1:80	1:100
Anti-SSA/52KD	+++	+++	+
Anti-dsDNA	–	–	–
Anti-SM	–	–	–
RF (IU/mL)	217	33.8	<10
Proteinuria (g/d)	0.11	9.78	0.055

ANA, antinuclear antibody; SM, smooth muscle; RF, rheumatoid factor; Follow-up, last follow-up (December 2024).

**Figure 1:**
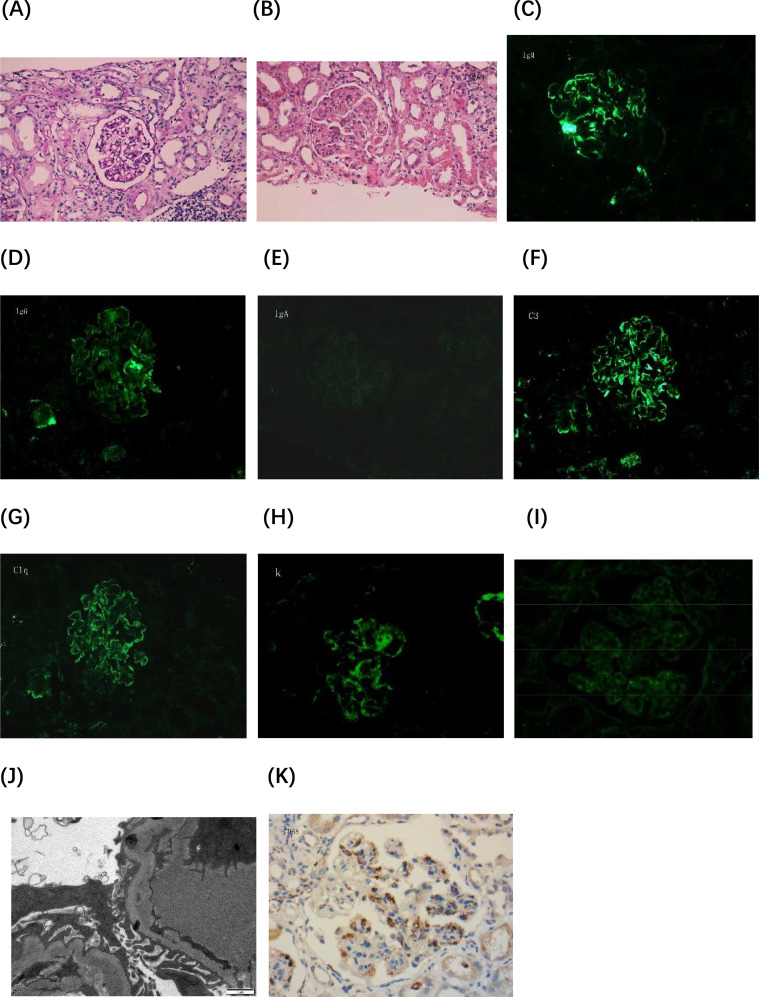
Histopathological evaluation of renal biopsy. **(A)** Periodic acid-Schiff (PAS) staining image 1 shows mild proliferation of mesangial cells and matrix, without mesangial insertion or a double-track sign (×400). **(B)** PAS staining image 2 demonstrates diffuse proliferation of mesangial cells and matrix in the glomerulus, along with mesangial insertion and double-track sign formation. The glomerulus exhibits a lobulated appearance, and microthrombi are present in capillary loops (×400). **(C-I)** Immunofluorescence images: **(C)** IgM (×400), **(D)** IgG (×400), **(E)** IgA (×400), **(F)** C3 (×400), **(G)** C1q (×400), **(H)** Kappa light chain (×400), and **(I)** Lambda light chain (×400). **(J)** Electron microscopy image reveals clustered electron-dense deposits beneath the endothelium and microthrombus formation within capillary loops, demonstrating uniform density and no discernible substructures (×15,000). **(K)** Immunohistochemistry image shows strong CD68 positivity in the glomeruli (×400). Lambda, kappa, and CD68 staining were performed on paraffin-embedded sections.

In August 2022, a renal biopsy was performed, revealing 12 glomeruli, including one with glomerulosclerosis (**Figure 1**). The remaining glomeruli exhibited moderate to severe diffuse proliferation of mesangial cells and matrix. Nodular lobulated changes were observed in eight glomeruli, along with increased cellularity in segmental capillaries and mild neutrophil infiltration. Diffuse basement membrane thickening with a double-track appearance was present in eight glomeruli (66.7%). Three glomeruli (25%) exhibited only mild mesangial cell and matrix proliferation, without nodular lobulated changes. A strongly periodic acid-Schiff (PAS)-positive microthrombus was detected in one glomerular vascular loop. Renal tubular epithelial exhibited vacuolar and granular degeneration, along with multifocal lymphomonocytic infiltration. Mucinous degeneration and segmental fibrinoid necrosis were noted in the intima of small arteries, with luminal narrowing and occlusion. Immunofluorescence analysis revealed IgM (3+), IgG (1+), C3 (3+), C1q (2+), kappa (3+), lambda (1+); IgA findings were negative. Positive immunoreactive deposits were clustered in the mesangial area and granularly distributed along the capillary loops. Light microscopy findings were consistent with a diagnosis of immune complex-mediated MPGN. Immunohistochemistry showed strong CD68 positivity in the glomeruli (**Figures 1K**). Electron microscopy revealed mild to moderate mesangial cell and matrix proliferation, segmental endothelial cell proliferation, and deposition of block-shaped electron-dense material in the mesangial area and subendothelium of affected segments. Cryoglobulin deposits were identified in capillary loops without discernible substructure. Most of the loose layer within the basement membrane appeared slightly widened, with partial fusion of foot processes. Electron microscopy findings indicated MsPGN with endothelial cell damage. Based on the combined results of light and electron microscopy, a diagnosis of MPGN was established. Cryoglobulin testing revealed type II cryoglobulin positivity, along with polyclonal IgG-λ and IgG-κ and monoclonal IgM-κ. Cyclophosphamide and methylprednisolone were administered. The total cumulative dose of cyclophosphamide was 12g.

During follow-up in December 2024, no evidence of cryoglobulinemia was detected. Serum creatinine was 82.1 µmol/L, complement C3 was 1.04 g/L, complement C4 was 0.107 g/L, and IgG was 10.6 g/L. RF was <10 IU/mL, and 24-hour urinary protein quantification was 0.055 g/24h.

## Discussion and conclusion

The patient had a 2-year history of pSS without proteinuria or hematuria. After a pulmonary infection in July 2022, her ANA titer was 1:320, and renal biopsy revealed MPGN-like lesions (if classified as type IV LN, scoring 10 points). Simultaneous decreases in complement C3 and C4 suggested systemic lupus erythematosus (SLE). The critical factor influencing a diagnosis of SLE in this patient was whether the MPGN-like lesions could be classified as type IV LN. If not, the SLE diagnosis would not be substantiated. The patient displayed type II cryoglobulinemia positivity, and cryoglobulinemic GN also presents as MPGN. Cryoglobulinemia may contribute to decreased levels of complement C3 and C4 (10). Several factors did not support an SLE diagnosis. 1) The patient exhibited persistent anti-dsDNA, anti-SM, and anti-ribosomal P protein negativity after disease onset. This serological profile is a key distinguishing factor between SLE and pSS (11). 2) Renal immunofluorescence findings did not exhibit a “full house” pattern. 3) Electron microscopy did not reveal electron-dense material deposition in the subepithelial region, nor were tubuloreticular inclusions observed. 4) Immunofluorescence findings in LN are typically dominated by IgG. In this patient, IgM fluorescence intensity was substantially stronger than that of IgG, which is a key distinguishing feature between cryoglobulinemic GN and LN (8). Aside from symptoms of dry mouth and dry eyes, the patient did not exhibit clinical manifestations of SLE (e.g., leukopenia, thrombocytopenia, autoimmune hemolysis, joint pain, oral ulcers, rash, or discoid erythema). Therefore, an SLE diagnosis was not supported. Disease exacerbation was likely triggered by pulmonary infection, which further worsened cryoglobulinemia and led to cryoglobulinemic GN.

MPGN is the second most common renal disease associated with pSS (6). In primary MPGN, mesangial and capillary proliferation manifests as a diffuse, spherical change; in secondary MPGN, lesions are predominantly focal and segmental (13). Secondary MPGN, (e.g., type IV LN), may manifest as non-diffuse MPGN-like lesions (14). In this case, more than 50% of glomeruli exhibited MPGN-like lesions under light microscopy, whereas some showed only mild mesangial cell and matrix proliferation. The diagnoses based on light and electron microscopy were not entirely consistent. In the early stages of cryoglobulinemic GN, immune complex-mediated thrombi obstruct glomerular capillaries, and not all deposits exhibit defined substructures (12), which is a characteristic pathological features of cryoglobulinemic GN. In the present case, strongly PAS-positive microthrombi were detected in glomerular capillary loops, and electron microscopy confirmed the presence of cryoglobulins within capillary loops. Additionally, monocyte/macrophage infiltration in glomeruli is a hallmark of active cryoglobulinemic GN (6). Immunohistochemistry revealed strong CD68 positivity in glomeruli. Immunofluorescence analysis showed predominant IgM and kappa light chain expression, along with IgG, C3, C1q, and lambda positivity, indicating the presence of monoclonal IgM kappa in the blood. In cryoglobulinemic GN, electron-dense deposits are more commonly found within subendothelial zones and mesangial areas; subepithelial and intramembranous deposits are less frequently observed (12).

Exacerbation of the patient’s condition was likely triggered by pulmonary infection. A previous study demonstrated that pulmonary infections can substantially increase the EULAR Sjögren’s Syndrome Disease Activity Index score in patients with pSS (8). Furthermore, infection can elevate type II cryoglobulin levels in patients with chronic active hepatitis C, leading to cryoglobulinemic GN onset (9). After pulmonary infection, the patient in this case exhibited a considerable increase in RF levels, type II cryoglobulinemia positivity, and the onset of acute kidney injury and nephrotic syndrome. Renal biopsy confirmed the diagnosis of cryoglobulinemic GN. After anti-infective treatment, RF and urinary protein levels rapidly declined; complement C3, complement C4, and serum creatinine returned to normal. Infection may serve as a critical factor in exacerbating cryoglobulinemia and triggering cryoglobulinemic GN onset in patients with pSS. Previous literature has also reported cases of cryoglobulinemic GN caused by pulmonary infection (15). Clinicians should consider more aggressive monitoring and early anti-infective treatment for pSS patients who develop infections.

To our knowledge, this is the first reported case of non-diffuse MPGN with cryoglobulinemic GN secondary to pSS. Infection may play a pivotal role in exacerbating cryoglobulinemia and inducing cryoglobulinemic GN.

## Data Availability

The original contributions presented in the study are included in the article/supplementary material. Further inquiries can be directed to the corresponding author.
